# Differentiating Autonomic Denervation Dermatitis From Post-traumatic Eczema: A Case Series of Localized Eczematous Eruptions

**DOI:** 10.7759/cureus.89021

**Published:** 2025-07-29

**Authors:** Piyush Yadav, Sushantika Sushantika, Poonam Saini, Jyoti Sethi, Kartik Saini

**Affiliations:** 1 Dermatology, All India Institute of Medical Sciences, Rishikesh, Rishikesh, IND

**Keywords:** autonomic denervation dermatitis, eczema, skinted, surgery dermatitis, trauma

## Abstract

Autonomic denervation dermatitis is a newly introduced term that describes the occurrence of a scaly eczematous lesion at the site of incision of a previous surgery. The time lag between the incision and the occurrence of the eczematous lesion varies greatly, ranging from months to years. Autonomic denervation dermatitis, by definition, can occur at any site of previous surgery; however, a few sites are specially named, like surgery of the knee, injury to the infrapatellar branch of the saphenous nerve, and traumatic eczematous dermatitis (SKINTED) occurring after total knee arthroplasty. Autonomic denervation dermatitis is believed to occur due to the transection of dermal nerves, which pathophysiologically differentiates it from post-traumatic eczema, an inflammatory reaction presenting as eczematous lesions that develop at and around sites of mechanical, thermal, or chemical injury. Herein, we describe a series of five such cases and try to differentiate between autonomic denervation dermatitis and post-traumatic eczema.

## Introduction

Dermatitis can arise from various causes, including allergic, atopic, irritant, and stasis. Dermatitis due to nerve injuries and trauma leads to conditions like autonomic denervation dermatitis and post-traumatic eczema. Autonomic denervation dermatitis is a rare localized eczematous eruption that occurs around surgical incision sites and is often associated with cutaneous anesthesia or hypoesthesia. It typically results from iatrogenic nerve damage and autonomic disruption following surgery [1]. In contrast, post-traumatic eczema develops at and around the site of previous mechanical, thermal, or chemical injury and represents an inflammatory reaction. The lesions usually develop around and exactly over the traumatized skin, often following an isomorphic pattern. While the clinical presentation and treatment are largely similar in both conditions, they arise from entirely different pathophysiological processes.

In this case series, we describe five patients who presented with similar localized eczematous plaques. Through history and clinical examination, three cases were diagnosed as autonomic denervation dermatitis, developing after surgery, while the other two cases were identified as post-traumatic eczema, following recent trauma. Despite their comparable presentation, recognizing the different etiologies is essential for guiding treatment and educating the patient regarding the cause, avoidance of potential triggers, and avoidance of unnecessary investigations.

## Case presentation

These cases describe five male patients, each presenting with eczematous plaques associated with either postoperative scars or traumatic injuries, ultimately leading to diagnoses of autonomic denervation dermatitis or post-traumatic eczema.

**Figure 1:**
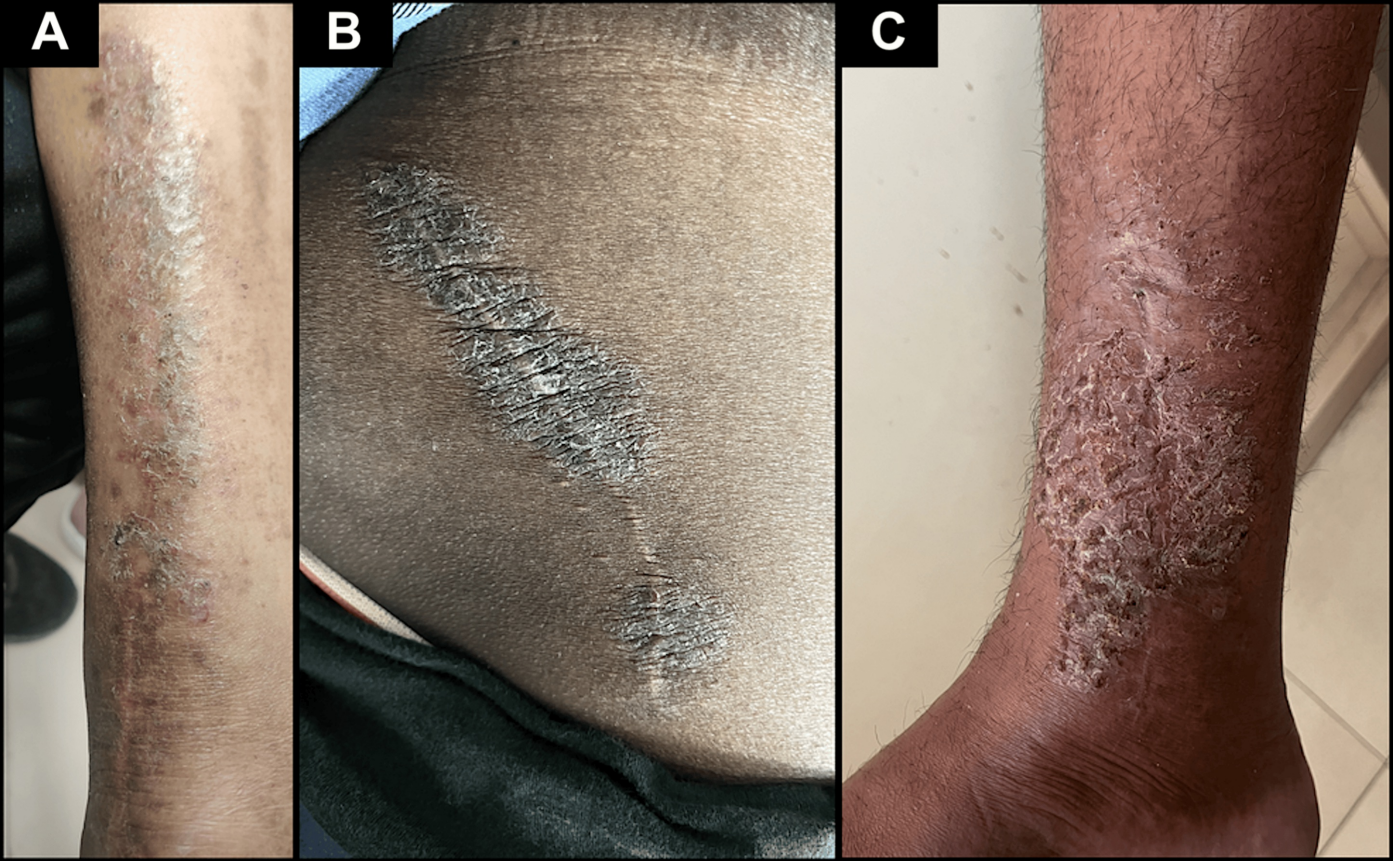
Lichenified plaque is present on and around the incision site, indicating xerosis and representing autonomic denervation dermatitis. Eczematous plaques observed in (A) Case one, (B) Case two, and (C) Case three.

Case one involves a 55-year-old male patient who presented with an eczematous plaque on his leg surrounding the postoperative scar from an open reduction and internal fixation of a left tibial fracture, which developed four months after the surgery (Figure 1A). Case two pertains to a 47-year-old male patient who presented with an eczematous plaque on a healed surgical scar from a total hip replacement surgery on the right side of his buttock, which appeared three years post-surgery (Figure 1B). Case three involves a 45-year-old male patient who presented with an eczematous plaque on his leg surrounding the postoperative scar from an open reduction and internal fixation of a right tibial fracture, which developed 2.5 months after the surgery (Figure 1C). The patient experienced decreased sensation to touch and diminished sweating in the affected area two weeks after the trauma.

**Figure 2:**
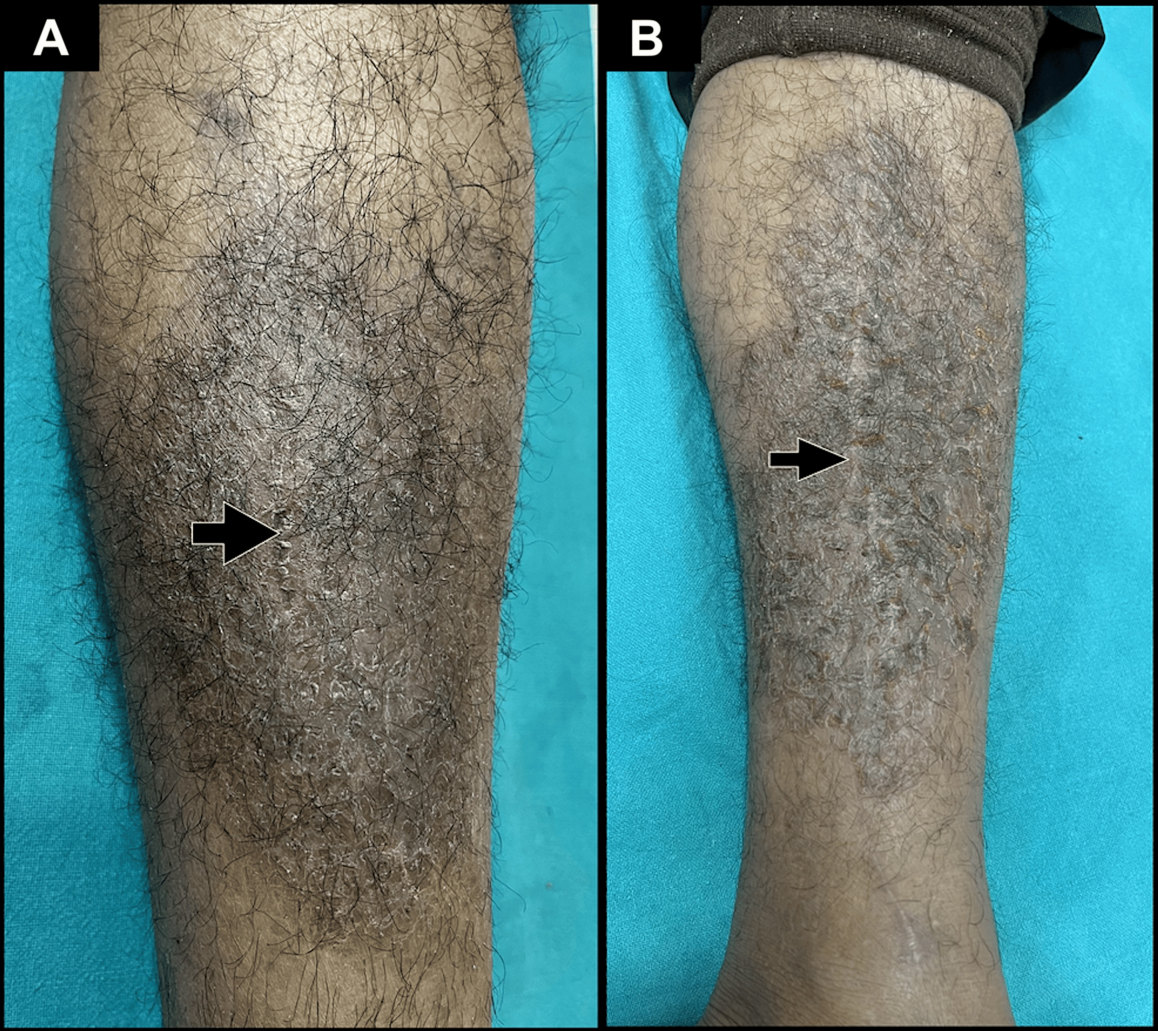
Lichenified plaque present on and around the scar site (black arrows) with a background of xerosis representing post-traumatic eczema. Lichenified plaques observed in (A) Case four and (B) Case five.

Case four involves a 34-year-old male patient who developed a similar itchy eczematous plaque on his right leg following a mechanical traumatic injury (Figure 2A). Case five pertains to a 50-year-old male patient who presented with a well-demarcated, hyperpigmented, oozing eczematous plaque located on the medial aspect of his left shin, with a history of mechanical trauma occurring five weeks prior to the appearance of the eczematous lesions (Figure 2B). The patient additionally provided a history of atopy.

No cutaneous lesions were observed in other areas of the body. Gram stain and potassium hydroxide examinations for fungal organisms yielded negative results. There were no signs and symptoms of venous stasis; therefore, venous Doppler was not done. The patients did not give consent for skin biopsy examination. Therefore, based on the clinical presentation and history, a diagnosis of autonomic denervation dermatitis was made in the first three cases and post-traumatic eczema in the remaining two cases. The patients were prescribed emollients and mild topical corticosteroids for local application, along with follow-up advice. Three patients were followed up after four weeks, showing complete or near-complete resolution of symptoms and lesions, while one patient did not return for follow-up. The patients did not attend subsequent follow-up visits.

## Discussion

The autonomic nerves play a significant role in maintaining normal skin barrier function and homeostasis. Autonomic nerve fibers, along with neurotransmitters like acetylcholine and catecholamines released from these nerve endings, innervate various structures in the dermis, including blood vessels, muscles, apocrine and eccrine glands, and hair follicles, thereby regulating their activity and maintaining normal skin barrier function [2,3]. Transection of autonomic nerves during surgery causes denervation of these cutaneous structures, leading to an alteration of the skin barrier and the initiation of a localized eczematous process. These skin lesions can develop on and beyond the wound sites.

Madke et al. first proposed the term "autonomic denervation dermatitis" in 2007 to describe all similar entities presenting as eczematous eruptions at and around the scar sites of previous surgeries [4]. SKINTED (surgery of the knee, injury to the infrapatellar branch of the saphenous nerve, traumatic eczematous dermatitis), first described by Verma and Mody in 2009 [5], which develops around the knee after knee arthroplasty, was included under the umbrella term autonomic denervation dermatitis. While SKINTED is a site- and procedure-specific eczematous eruption, autonomic denervation dermatitis includes all similar entities. "Neuropathy dermatitis" is a nonspecific and misleading term that falls under the umbrella, as it lacks the other features of sensory or motor neuropathy typically associated with this condition [6]. Madke et al. also observed that the rash selectively appeared only on the surgical sites of the lower limb and not on the chest scar following coronary artery bypass grafting. This was attributed to the relatively poor blood supply in the lower limb, which is further compromised by autonomic nerve damage.

Post-traumatic eczema is a similar entity, presenting as eczematous lesions that develop at and around sites of mechanical, thermal, or chemical injury. However, post-traumatic eczema is different from autonomic denervation dermatitis in many ways, as highlighted in Table 1 [4,6,7]. The mechanism behind the development of these eczematous lesions is poorly understood but is thought to be an inflammatory response following trauma. Atopic diathesis has been implicated as an underlying cause in the development of post-traumatic eczema. Lesions of post-traumatic eczema frequently recur over many years (up to eight years) [4].

**Table 1:** Differences between autonomic denervation dermatitis and post-traumatic eczema. Table modified from [4].

	Autonomic denervation dermatitis	Post-traumatic eczema
Site	Occurs on the post-surgical site	Occurs on the post-traumatic site
Type of trauma	Surgical	Mechanical/thermal/chemical
Onset	Months to years	Within 3–4 weeks
History of atopy	Absent	Present
Pathophysiology	Transection of the dermal nerves causing autonomic disturbance	Inflammatory response to trauma
Recurrence	Persistent or recurrent	Recurrent

Treatment involves the liberal use of emollients and occlusive moisturizers to maintain skin barrier integrity. Since autonomic denervation dermatitis is a steroid-responsive dermatosis, an appropriate-strength topical corticosteroid is the treatment of choice. The clinical course of autonomic denervation dermatitis is not well understood and may involve a long-term persistent eczematous rash and periods of remission and relapse, particularly in winter. Proper patient counseling is essential to address the chronicity of the disease, and the liberal use of emollients should be emphasized to the patient.

In terms of preventing autonomic denervation dermatitis, the aim of the treating surgeon should be to minimize the iatrogenic injury to the nerve, which initiates this cascade, leading to the development of autonomic denervation dermatitis plaques. Intraoperative steps to minimize the occurrence of SKINTED have been described by previous authors, including the anterolateral skin incision approach and using a modified oblique incision for hamstring graft harvesting, among others [8]. Prevention of post-traumatic eczema involves avoiding triggers such as physical traumas (burns, surgery, and insect bites), mechanical irritation (tight clothing, medical tape, and chronic friction), and chemical irritation (harsh wound-care products, soaps, and detergents). Identifying and avoiding these triggers can aid in preventing post-traumatic eczema, especially in individuals with atopic predisposition.

Our case series is subject to several limitations, notably a restricted sample size, the inability to conduct skin biopsies, and the absence of long-term follow-up data.

## Conclusions

Distinguishing between autonomic denervation dermatitis and post-traumatic eczema can be challenging due to their overlapping clinical presentations. Recognizing these differences helps clinicians reassure patients, choose appropriate therapy, and prevent unnecessary investigations. Our case series has highlighted the importance of clinical history and physical examination to differentiate the two similarly presenting entities. Autonomic denervation dermatitis is a relatively new term that is relevant to both dermatologists and surgeons. Greater awareness among clinicians and further studies are required to fully understand this condition.
